# The Temporal Order of Mixed Viral Infections Matters: Common Events That Are Neglected in Plant Viral Diseases

**DOI:** 10.3390/v16121954

**Published:** 2024-12-20

**Authors:** Celia de Moya-Ruiz, Inmaculada Ferriol, Pedro Gómez

**Affiliations:** 1 Departamento de Biología del Estrés y Patología Vegetal, Centro de Edafología y Biología Aplicada del Segura (CEBAS)-CSIC, C.P. 30100 Murcia, Spain; cmoya@cebas.csic.es; 2 Instituto de Ciencias Agrarias (ICA)-CSIC, C.P. 28006 Madrid, Spain; iferriol@ica.csic.es

**Keywords:** CABYV, Co-infection, mixed infections, sequential infection, virus–virus interaction, within-host virus interaction, WMV

## Abstract

Mixed infections of plant viruses are common in crops and represent a critical biotic factor with substantial epidemiological implications for plant viral diseases. Compared to single-virus infections, mixed infections arise from simultaneous or sequential infections, which can inevitably affect the ecology and evolution of the diseases. These infections can either exacerbate or ameliorate symptom severity, including virus–virus interactions within the same host that may influence a range of viral traits associated with disease emergence. This underscores the need for a more comprehensive understanding of how the order of virus arrival to the host can impact plant disease dynamics. From this perspective, we reviewed the current evidence regarding the impact of mixed infections within the framework of simultaneous and sequential infections in plants, considering the mode of viral transmission. We also examined how the temporal order of mixed infections could affect the dynamics of viral populations and present a case study of two aphid-transmitted viruses infecting melon plants, suggesting that the order of virus arrival significantly affects viral load and disease outcomes. Finally, we anticipate future research that reconciles molecular epidemiology and evolutionary ecology, underlining the importance of biotic interactions in shaping viral epidemiology and plant disease dynamics in agroecosystems.

## 1. Introduction

Plant viruses represent a serious threat to agriculture due to the lack of effective countermeasures to control their diseases. Within these plant viral diseases, mixed infections are common in crops and are increasingly recognized as an integrated biotic factor that can affect the ecology and evolution of epidemics. This complex of infections in the same host can occur in all living domains, from bacteria [1,2] and fungi [3] to plants [4,5,6], animals [7,8], and humans [9] by different types of parasite microbes. Such mixed infections may occur by either unrelated [6,10,11] or related pathogens [7,12,13,14], with important socio-economic impacts. For example, mixed infections with bacteria and viruses are very common in humans, deteriorating immunological functions and increasing the risk of morbidity and mortality [15,16], such as in the case of *Mycobacterium tuberculosis* and *Lentivirus humimdef1* (human immunodeficiency virus 1, HIV-1) [17]. In addition, co-infections between fungi and viruses have recently been shown in plants, where, for the fungus *Phomopsis subordinaria*, transmission increases despite its restriction in the infection rate when co-infecting with the virus *Capulavirus plantagonis* (plantago lanceolata latent virus, PILV) [6]. Similarly, co-infections have also been observed between viruses; *Betacoronavirus pandemicum* (severe acute respiratory syndrome coronavirus 2, SARS-CoV-2) and other respiratory viruses such as *Influenza A virus*, rhinovirus/enterovirus, para-influenza, metapneumovirus, and *Influenza B virus* [18], with similar relevance in plants, whereas virus-virus combinations within the same plant have been described to cause major losses in crops [19,20].

Within the agricultural context, many factors may influence the occurrence of multiple viral infections in the same plant and crop. For example, both viral and plant intrinsic factors, such as generalist viruses, plant species, cultivar, age, and nutritional status, as well as external factors, such as the mode of virus transmission, polyphagous vectors, environmental conditions, growing season overlap, intensification and expansion of agricultural production, and proximity to alternative hosts, are thought to occur in agricultural contexts and are likely to combine, leading to a range of ecological interactions between plants and viruses that may favor the occurrence and prevalence of mixed infections. Additionally, viral disease management based on visual inspections can result in inefficiency, as mixed infections may allow either misinterpretation of symptoms during monitoring or even non-detection of the focal virus [21], with unpredictable epidemiological and important socio-economic consequences [20,22,23,24,25,26,27]. In this sense, virus–virus interactions within the same host have been described and traditionally defined as synergistic, neutral, and antagonistic, and more details on these interactions have been addressed in recent reviews [20,28,29]. Briefly, facilitating interactions or synergism can occur when mixed infections lead to increased replication of one or both viruses or more severe symptomatology compared to single infections. Such synergistic effects may be attributed to various mechanisms, including the suppression of host defense responses by one virus, which inadvertently benefits the other, or the complementary exploitation of host resources by different viral species [20,23,28,30]. On the other hand, antagonistic interactions between viruses may manifest as reciprocal exclusion, wherein both viruses experience a decline in replication when infecting together, or where the infection of one virus leads to the suppression of another, possibly due to competition for limited cellular resources or the activation of broad-spectrum host defense mechanisms [20,28,29,31,32,33]. These virus–virus interactions within the same host plant are believed to exert significant selection pressures on viral populations, potentially driving their evolutionary trajectories in ways that differ from those observed in single infections [29]. However, the long-term implications of these interactions on viral evolution and disease dynamics remain unclear, requiring further investigation to fully elucidate their complex interplay and ecological consequences.

The occurrence of mixed viral infections in crops indicates that multiple transmission events occur in the same plant, and these infections arise from either simultaneous infections (co-infections) or sequential infections (one after the other). Within this framework, and considering that most plant viruses are transmitted by insect vectors [34], it is postulated that the temporal order of the arrival of different viruses on the same plant can inevitably affect the ecology and evolution of viral diseases. In this article, we review the current evidence and highlight the importance of mixed infections in plants, contingent upon the temporal order of viral transmission. By examining co- and sequential infections of two virus species that infect melon plants, we provide insights into the complex dynamics of viral transmission, indicating that the order of viral infections in plants is significant in viral load. We then considered how the temporal order of viral infections could shape the population and evolutionary dynamics of plant viruses, emphasizing the necessity of incorporating biotic factors to gain a better understanding of the ecological mechanisms that drive viral epidemiology.

## 2. The Insect-Vector Role in Mixed Infections of Plant Viruses

**Figure 1:**
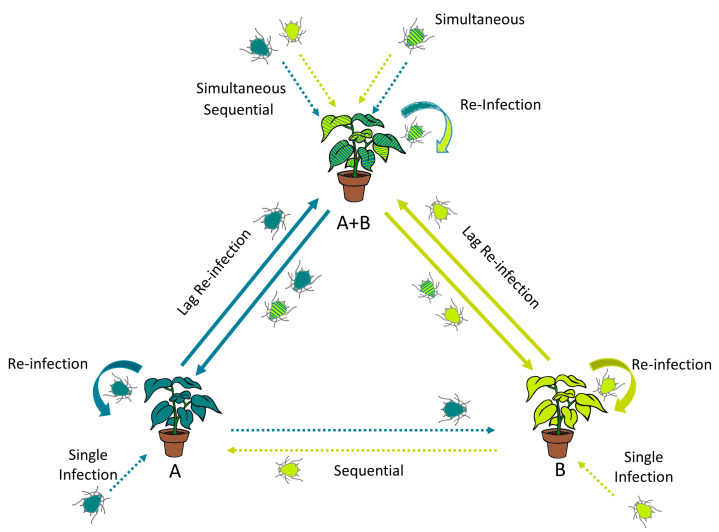
Outline of potential scenarios for generating mixed infections in cultivated plants. Assuming that either cultivated or wild plant species, including secondary plant structures (non-crop plants utilized in integrated pest management) within the crops, can serve as a source of inoculum and that both viruses share an insect vector-mediated transmission route, mixed infections may be generated by simultaneous or sequential infection processes from single or co-infected plants (dotted lines), with potential subsequent re-infections (solid lines). Insect vectors are colored differently according to the viral source plant (single or mixed infection) where they are fed.

A significant percentage of plant viruses (>70%) is spread by insect vectors of the order Hemiptera, such as aphids, whiteflies, and leafhoppers [35,36], which are also known to be pests on cultivated plants in temperate regions. Additionally, beetles, thrips, mealybugs, fungi, and nematodes are key vectors for the transmission of some viral species, including human assistance [37]. Viral transmission by insects has been classically classified as non-persistent, semi-persistent, or persistent, and some reviews have dealt with these aspects in depth [34,38,39,40,41]. While non-persistent viruses have short retention times in their vector, with acquisition and inoculation access periods also short over hours, persistent viruses can be retained for longer periods, with acquisition and inoculation periods over several days, versus semi-persistent viruses spending an intermediate time between the previous ones [34,42]. With these time periods, and contingent upon the mode of viral transmission, insect vectors can transmit multiple viruses, either from the same host with mixed infections (co-transmission) or from different hosts with different viral diseases in a sequential order (Figure 1).

Regression analyses of multispecies plant-virus system networks showed that co-infections were the most frequent mode of infection in the ecosystem and contributed more to ecological interactions than single infections [43]. However, how co-infections and sequential infections influence the prevalence of mixed viral infections is still poorly understood in plants. For example, studies on *Orthoflavivirus denguei* (dengue virus, DENV), *Orthoflavivirus zikaense* (zika virus, ZIKV), and *Alphavirus chikungunya* (chikungunya virus, CHIKV), which are transmitted by *Aedes aegypti* and *Aedes albopictus*, indicate that the co-circulation of these viruses enhances both consecutive and simultaneous infections. In some regions of South America, the prevalence of DENV/CHIKV co-infection has reached 10%**,** along with the occurrence of DENV/ZIKV and DENV/ZIKV/CHIKV combinations [44,45]. In this context, models of transmission dynamics suggest that a majority of coinfections would result from sequential bites from different mosquitoes carrying different viruses rather than co-transmission [46]. Although, if the probability of co-transmission is high, the model indicates that majority of coinfections could be attributed to co-transmission, and the prevalence of coinfections would be more than double what would be expected from sequential infections. In plants, mixed viral infections are widespread. For example, our previous studies on the prevalence of aphid-borne viral diseases affecting cucurbit plant species reveal that mixed infections occur in a high frequency in these crops. A significant proportion (20–40%) of symptomatic samples exhibit co-detection predominantly involving the *Polerovirus CABYV* (cucurbit aphid-borne yellows virus, CABYV) and *Potyvirus citrulli* (watermelon mosaic virus, WMV), including the emerging *Polerovirus PABYV* (pepo aphid-borne yellows virus, PABYV). Additionally, mixed infections have been detected involving viruses belonging to the genus *Potyvirus*, such as *Potyvirus cucurbitaflavitesselati* (zucchini yellow mosaic virus, ZYMV), *Potyvirus papayanuli* (papaya ringspot virus, PRSV), and *Potyvirus citrullimoroccense* (Moroccan watermelon mosaic virus, MWMV) [21,24,47,48,49]. With the expected rise in insect vector populations in crops, it is likely that the frequency of mixed infections will even increase during the next seasons. However, it is unclear to what extent the order and timing of these cucurbit virus infections can influence virus–virus interactions within the same host and transmission dynamics, and, although these insect transmissions can be dependent on the environmental conditions, insect-feeding behavior, and agricultural practices, further empirical and modeling research could predict plant virus epidemiology.

Given that transmission rates become a critical factor influencing disease dynamics, when multiple viruses infect the same plant, the viral load of each virus can ultimately influence which virus prevails within the populations. This viral competition for transmission is more likely to occur when different viruses share the same insect vector species to spread from plant to plant, since viruses can interact with each other, affecting their individual viral loads and transmission dynamics [50]. A vector transmission modeling study has highlighted the importance of vector competition as a critical factor in co-infections, emphasizing its role in shaping the outcomes of mixed infections [51]. For instance, studies on the co-transmission of different *Potyvirus yituberosi* strains (potato virus Y, PVY) by aphids have shown that a single aphid can simultaneously acquire and transmit more than one PVY strain. This could occur either by acquiring both strains from a single infected plant or by sequential acquisition from different sources [52,53,54]. However, the transmission efficiency was found to be lower compared to single-strain transmission, indicating potential competition between the viruses during the transmission process. Focusing on aphid-transmitted viruses, research has predominantly focused on studies of simultaneous infections and the types of interactions that occur between these viruses [20], while few studies have considered the sequential order of infection by non-related viruses (Table 1). Among them, studies include the PVY/*Cucumovirus CMV* (cucumber mosaic virus, CMV) system in tomato, CMV/*Potyvirus capsimaculae* (pepper mottle virus, PepMoV) in pepper, and *Potyvirus betaceum* (beet mosaic virus, BTMV) with *Polerovirus BCHV* (beet chlorosis virus, BChV), *Polerovirus BMYV* (beet mild yellowing virus, BMYV) or *Closterovirus flavibetae* (beet yellows virus, BYV) in sugar beet, which highlight the interactions between viruses under these specific conditions [55,56,57].

Overall, Table 1 highlights that most interactions involving simultaneous or sequential virus infections result in synergism, with increased viral titers and enhanced symptom severity, regardless of the transmission mode. Non-persistent viruses appear to rely more on plant-host synergism, as these viruses typically localize to specific stylet sites, enabling rapid and transient transmission that likely precludes direct interactions within the insect. Persistent viruses may interact within both vectors and plant hosts, offering greater opportunities for direct interactions with co-infecting viruses. As mentioned before, with the anticipation that the occurrence of mixed infections may increase, along with the increase in aphid populations in crops, future research should prioritize investigating the impact of mixed infections on disease dynamics, viral interactions, and evolutionary outcomes in aphid-transmitted viral systems.

## 3. Impact of Temporal Order of Infection on Plant Viral Disease: A Case Study of Two Aphid-Transmitted Viruses

**Figure 2:**
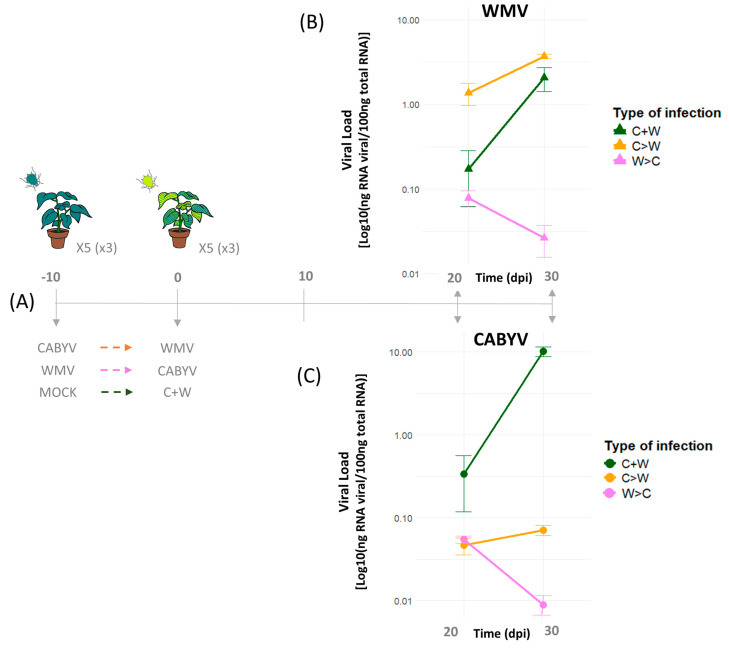
Viral load dynamics in single, co-, and sequential infections of CABYV and WMV in melon plants. (**A**) Schematic diagram of the experimental design for the infection of melon plants with WMV-MeWM7 and CABYV-LP63 by *Aphis gossypii*. Plants were inoculated with either CABYV or WMV, both viruses (simultaneous co-infection, C + W, green color), or mock treatment (control) on day −10. On Day 0, sequential infections were initiated: plants initially infected with CABYV were inoculated with WMV (C > W, orange color), and plants initially infected with WMV received CABYV (W > C, pink color). The RNA viral load (Log10 (ng of viral RNA/100 ng of total RNA) mean and SE error bars, n = 3) of WMV (**B**) and CABYV (**C**) in melon plants under simultaneous and sequential infections were determined by absolute quantification using RT-qPCR with specific RNA transcripts generated from the P1 and CP cloned genes of WMV and CABYV, respectively [24,49]. These in vitro RNA transcripts were serially diluted (10-fold) to generate external standard curves. The RNA concentration was estimated from the threshold cycle (CT) values obtained from three independent plant samples, with three technical replicates for each sample at each time point (20 and 30 dpi with aphids). Statistical comparisons were performed using linear mixed model fit by REML (*t*-tests use Satterthwaite’s method) to assess significant differences in viral loads between infection conditions over time.

To address how the temporal viral arrival order can impact virus accumulation and disease progression, it is advisable to conduct appropriate assays that enable controlled manipulation of simultaneous or sequential viral infections by insect vectors. This necessitates the utilization of viral infectious clones and aviruliferous insects to synchronize viral inoculations in plants at different temporal intervals. However, there is a paucity of studies elucidating the impact of mixed infections, particularly with the aphid-vector, *Aphis gossypii* Glover. In this sense, we carried out an experimental assay in which the viral load of two aphid-transmitted viruses; CABYV (persistent transmission mode) and WMV (non-persistent transmission mode), was examined in melon plants (Piel de Sapo cultivar) under different order of infections. By using CABYV and WMV infectious clones [24,49], both viral isolates were manipulated to obtain viruliferous aphids (*A. gossypii*) for subsequent melon plant infections either simultaneously or sequentially (one after the other), with a time lag of 10 days. Viral accumulation was estimated at 20 and 30 days post-inoculation (dpi) in co-infected and sequentially infected plants by RT-qPCR (Figure 2A). Our results showed that co-infection led to the highest CABYV and WMV accumulation, suggesting a double-synergistic effect when both viruses infect simultaneously (Figure 2B–C). Consistent with our results, a study of co-infection with *Fijivirus boryzae* (southern rice black-streaked dwarf virus, SRBSDV) and *Oryzavirus oryzae* (rice ragged stunt virus, RRSV) showed an increase in both viral titers as a consequence of changes in virus-induced RNAi pathway genes [73]. Similarly, a synergistic interaction was observed between *Potyvirus glycitessellati* (soybean mosaic virus, SMV) and two comoviruses, *Comovirus siliquae* (bean pod mottle virus, BPMV) and *Comovirus vignae* (cowpea mosaic virus, CPMV), regardless of whether the infection was simultaneous or sequential, with higher titers of BPMV and CPMV [74]. In sugar beet plants, co-infection of the potyvirus BTMV with either the closterovirus BYV or the polerovirus BWYV demonstrated a synergistic interaction between both viruses involved, leading to increased viral accumulation and more severe symptoms in the plants [58]. Other studies on simultaneous infections showed a type of synergistic-neutral interaction, where one virus increases its viral titer while the other remains unaffected, such as the interaction between *Orthotospovirus tomatomaculae* (tomato spotted wilt virus, TSWV) and ToCV in tomato plants [75], and PVY/CMV or CMV/PepMoV [61,63]. Such interactions can often be explained by viral complementation, a form of co-evolutionary synergism between different viruses during mixed infections. In this context, one virus can enhance the infection of another through mechanisms such as genetic exchange, reassortment, or trans-complementation, where viral proteins from one virus support the infection process of the other [28]. For instance, PVY has been shown to complement CMV mutant in accessing tomato phloem elements [56]. Similarly, interactions between *Potexvirus cymbidii* (CymMV) and *Tobamovirus odontoglossi* (ORSV) showed complementation of MPs and CPs to facilitate their movement within plants [76]. In contrast, other studies have shown that co-infection of *Nicotiana tabacum* with potexvirus *Potexvirus ecspotati* (potato virus X, PVX) and potyvirus PVY causes antagonistic interactions [77], which could reduce the symptoms of viral disease. Another study on *Tobamovirus mititessellati* (tobacco mild green mosaic virus, TMGMV) and oilseed rape mosaic virus (ORMV) tobamoviruses in tobacco plants showed that co-inoculated plants at the same time showed antagonistic interactions among these viruses as well as in sequential infection regardless of which virus arrived first in the plant [78]. This suggests that, while some particular virus species maintain a neutral interaction in mixed infections, either in simultaneous or sequential infections, other cases involve potential changes due to the direct virus–virus interactions or indirect host-response modulation, which could even be influenced by environmental factors [29,79].

Furthermore, simultaneous infection of sugar beet plants with BTMV and BYV showed a synergistic interaction [58], while sequential infection with BTMV followed by BYV ten days later resulted in a neutral interaction [57]. In the case of sequential CABYV and WMV infections, our results showed that the initially infecting virus appears to significantly affect the subsequent virus infection in melon plants. Upon analysis of the WMV load, we found that when WMV was introduced first, WMV reduced its viral load over time (*t*(*19*) = −2.549, *p* = 0.019607), indicating potential interference or antagonistic interaction with CABYV (Figure 2B). However, when CABYV arrived first, there was an increase in the viral accumulation of WMV, which was comparable to that in co-infections (*t*(*19*) = 3.359, *p* = 0.003296). In contrast, analysis of the CABYV load revealed that when WMV was infected first, there was a significant decrease in CABYV accumulation (*t*(*19*) = −2.778, *p* = 0.0120) while there was a minimal increase in CABYV when CABYV arrived before WMV (*t*(*19*) = −2.764, *p* = 0.0124) (Figure 2C). These results suggest that the temporal order of infection affects the type of interaction between these two viruses, with a synergistic interaction occurring when CABYV precedes WMV, and an antagonistic interaction when WMV precedes CABYV. These results were consistent with the case of mixed infections between PRSV and *Potexvirus papayae* (papaya mosaic virus, PapMV) in papaya plants, wherein simultaneous infections or sequential infections with PRSV followed by PapMV exhibited more severe symptoms than sequential infections with PapMV followed by PRSV [77]. Indeed, evidence suggests that antagonism results from the activation of innate and adaptive immunity, with elevated ethylene levels as well as RNAi-mediated resistance, in contrast with synergistic interactions [80]. Consequently, these differences in the host-mediated response underscore the complex dynamics of mixed infections, which necessitates consideration not only of these virus–virus interactions within the host to elucidate epidemiological patterns observed in crops [24,49,81], but also of the potential role of abiotic factors and feeding behavior of insect vectors in generating the context for mixed infections.

## 4. Consequences of Infection Order on Population Dynamics, Virus Transmission, and Agroecosystems

The temporal order of viral infection can significantly impact plant health. In certain instances, it may be beneficial to the host, as previous infection of an attenuated virus isolate may prevent subsequent infection by a virulent strain, a phenomenon known as cross-protection [82,83,84,85]. However, mixed infections are frequently detrimental, and regardless of the infection timing and focusing on insect vector-mediated transmission of plant viruses, within-host competition between viruses is not necessarily beneficial and could even facilitate an increased transmission and dispersion of the disease [70]. For instance, it has been observed that the variant zucchini yellow mosaic virus (ZYMV-WK) is transmitted by aphids from WMV-co-infected plants [86,87], with variations observed from other vectors [88]. Also, *Begomovirus capsicumhuastecoense* (pepper huasteco yellow vein virus, PHYVV) and *Begomovirus capsicummusivi* (pepper golden mosaic virus, PepGMV) can be co-transmitted by the whitefly *Bemisia tabaci* to pepper plants with no competition [89], similar to the co-transmission of Mld- and Il-strains of tomato yellow leaf curl virus (TYLCV) to tomato [90]. Another study showed that *Crinivirus cucurbitae* (cucurbit yellow stunting disorder virus, CYSDV) and WMV were transmitted by their corresponding vectors, whiteflies and aphids, respectively, without competition in transmission [91]. However, the co-infection of TYLCV and tomato mottle virus (ToMoV) by *Bemisia* to tomato plants showed competition in transmission, including differences in the infection status of plants and virus accumulation [92]. Likewise, co-infection of *Begomovirus cucurbitae* (cucurbit leaf crumple virus, CuLCrV) and CYSDV resulted in a lower CYSDV accumulation and reduced viral titer in whiteflies [93]. In contrast, the co-infection of BChV and BYV only reduced transmission of BChV, despite similar virus accumulation [94]. Furthermore, the transmission of *Polerovirus PEVYV2* (pepper vein yellows virus-2, PeVYV-2) and *Polerovirus PEWBVYV* (pepper whitefly borne vein yellows virus, PeWBVYV) by aphids and whiteflies, respectively, showed significant differences between simultaneous and sequential co-infections [95]. In addition to the viral transmission rate and accumulation, mathematical models have demonstrated that the vector’s preference for the host, the virus transmission mode, and the vector phenotype can influence vector population density, thereby having a significant impact on the disease incidence [96]. In fact, it has been shown that the timing of infection and aphid-mediated inoculation density significantly impact disease development, which is essential for establishing economic control thresholds in decision-support systems [57]. Consequently, it is necessary to ascertain whether viral co-transmission contributes to simultaneous mixed infections or if, alternatively, sequential infections are more prevalent, thereby indicating a lower frequency of co-transmission in crops, which could affect virus virulence and population dynamics.

Despite the significant impact of plant viruses on crops, wild plant species may serve as reservoirs and sources of inoculum, contributing to the spread of viral diseases in agricultural systems [97]. An increasing number of studies in wild plants have revealed that the prevalence of viral infections varies widely depending on the virus and host [4,98,99,100,101]. However, the occurrence of mixed infections in wild plants and their impact on crops and ecosystems remain poorly understood. Some studies indicate that virus prevalence and infection rates in wild plants exhibit greater variability than those in agricultural systems, likely attributed to the genetic diversity of wild plants [102]. Notably, viruses found in wild plants are often detected in cultivated crops [103,104,105], suggesting that wild plants may play an important role in generating mixed viral infections in crops. Additionally, global warming and climate change are likely to influence not only insect vector populations, thereby affecting the prevalence of mixed viral infections, but also crop productivity. A recent study has shown the detrimental effects of drought on melon plants, particularly in the context of mixed viral infections, which can further complicate plant health and crop productivity [67]. Herein, while water stress negatively affected vegetative growth and led to a higher proportion of female flowers in drought-stressed plants compared to controls, this increase resulted in a higher rate of fruit abortion, suggesting that the combination of drought and co-infections (CABYV and CMV) may enhance fruit abortion rates, and, consequently, reduce crop productivity [67]. Given that global warming is anticipated to increase the incidence of viral diseases, the opportunities for mixed infections will subsequently increase. Therefore, further research in the performance of crops bred for drought or heat tolerance, along with mixed viral infections, is crucial to understand the complex relationships between viruses, their vectors, and host plants, including environmental changes in order to facilitate more effective disease management strategies in agriculture and natural ecosystems.

## Figures and Tables

**Table 1:** Experimental studies on unrelated aphid-transmitted viruses in sequential and simultaneous infections.

Infection Type	Genus	Virus	Host	Interaction	Viral Trait	Cite
Sequential	*Potyvirus* */*Cucumovirus* *	PVY/CMV	Tomato	Synergism	Viral titer and symptoms	[56]
	*Potyvirus* */*Polerovirus* ▫	BTMV/BChV	Sugar beet	Neutral	Symptoms	[57]
	*Potyvirus* */*Polerovirus* ▫	BTMV/BMYV	Sugar beet	Synergism	Symptoms	[57]
Sequential and simultaneous	*Potyvirus* */*Polerovirus* ▫	WMV/CABYV	Melon	Synergism	Viral titer	This study
	*Potyvirus* */*Closterovirus* †	BTMV/BYV	Sugar beet	Neutral/Synergism	Symptoms and viral titer	[57,58]
	*Cucumovirus* */*Potyvirus* *	CMV/PepMoV	Pepper	Synergism	Viral titer and symptoms	[55]
Simultaneous	*Cucumovirus* */*Potyvirus* *	CMV/BICMV ^1^	Cowpea	Synergism	Viral titer and symptoms	[59,60]
	*Cucumovirus* */*Potyvirus* *	CMV/PVY	Tobacco	Synergism	Viral titer and symptoms	[61]
	*Cucumovirus* */*Potyvirus* *	CMV/TuMV ^2^	*Nicotiana benthamiana*	Synergism	Symptoms	[62]
	*Cucumovirus* */*Potyvirus* *	CMV/PepMoV	Pepper	Synergism	Viral titer and symptoms	[63]
	*Cucumovirus* */*Potyvirus* *	CMV/WMV	Zucchini squash and melon	Synergism	Viral titer and symptoms	[64]
	*Cucumovirus* */*Potyvirus* *	CMV/ZYMV	Bottle gourd, Zucchini squash and melon	Synergism	Viral titer and symptoms	[64,65,66]
	*Cucumovirus* */*Polerovirus* ▫	CMV/CABYV	Melon	Synergism	Symptoms	[67]
	*Potyvirus*/*Caulimovirus* †	TuMV/CaMV ^3^	*Arabidopsis thaliana*	Neutral	Viral titer and symptoms	[68]
	*Potyvirus* */*Cucumovirus* *	TEV ^4^/CMV	*N. benthamiana*	Synergism	Viral titer and symptoms	[69]
	Potyvirus */*Polerovirus ▫*	PVY/PLRV ^5^	Potato	Synergism	Viral titer and symptoms	[70]
	Potyvirus */*Polerovirus ▫*	BTMV/BWYV ^6^	Sugar beet	Synergism	Symptoms and viral titer	[58]
	Potyvirus */*Polerovirus ▫*	PVY/PLRV	*N. clevelandii*	Synergism	Viral titer	[71]
	Polerovirus */*Umbravirus ▫*	TuYV ^7^/CMoV ^8^	*N. benthamiana*	Synergism	Viral titer	[72]

1 Blackeye cowpea mosaic virus, BICMV 2 *Potyvirus rapae* (turnip mosaic virus, TuMV) 3 *Caulimovirus tessellobrassicae* (cauliflower mosaic caulimovirus, CaMV) 4 *Potyvirus nicotianainsculpentis* (tobacco etch virus, TEV) 5 *Polerovirus PLRV* (potato leafroll virus, PLRV) 6 *Polerovirus BWYV* (beet western yellows virus, BWYV) 7 *Polerovirus TUYV* (turnip yellows virus, TuYV) 8 *Umbravirus maculacarotae* (carrot mottle virus, CMoV). Transmission modes: ▫ Persistent; † semi-persistent; and * non-persistent.
